# Decreased Left Ventricular Mass is Associated with Sarcopenia and its Severity in Elderly Inpatients

**DOI:** 10.5334/gh.1326

**Published:** 2024-05-06

**Authors:** Yang Liu, Ling Li, Hui Gong, Xing Lyu, Lini Dong, Xiangyu Zhang

**Affiliations:** 1 Department of Geriatrics, The Second Xiangya Hospital, Central South University, Changsha, Hunan, CN; 2 Department of Clinical laboratory Medicine, The Second Xiangya Hospital, Central South University, Changsha, Hunan, CN; 3 Hunan Clinical Medical Research Center for Geriatric Syndrome, Changsha, Hunan, CN

**Keywords:** skeletal muscle mass, left ventricular mass, sarcopenia, bioelectrical impedance analysis, handgrip strength

## Abstract

**Objective:**

Skeletal muscle mass and cardiac structure change with age. It is unclear whether the loss of skeletal muscle mass (SMM) is accompanied by a decrease in heart mass loss. The aim of this study is to investigate the relationship of left ventricular mass (LVM) with sarcopenia and its severity in elderly inpatients.

**Methods:**

Seventy-one sarcopenia subjects and 103 non-sarcopenia controls were enrolled in this study. Bioelectrical impedance analysis, handgrip strength, and 5-time chair stand test were used to evaluate SMM, muscle strength, and physical performance, respectively. Myocardial structure and function were assessed by echocardiography. Sarcopenia was diagnosed according to the Asian Working Group for Sarcopenia criteria 2019.

**Results:**

Sarcopenic patients had smaller left ventricular sizes and LVM than non-sarcopenic controls. Severe sarcopenic patients had smaller left ventricular sizes and LVM than non-severe sarcopenic patients. In univariate regression analysis, body mass index (BMI), cardiac size, and LVM were positively correlated with SMM or SMI. In multivariate regression analysis, BMI and LVM were independently correlated with SMM and SMI. The combined measurement of LVM and BMI predicts sarcopenia with 66.0% sensitivity and 88.7% specificity (AUC: 0.825; 95% CI: (0.761, 0.889); *p* < 0.001).

**Conclusion:**

In hospitalized elderly patients, decreased left ventricular mass is associated with sarcopenia and its severity, and the combined measurement of LVM and BMI has a predictive value for sarcopenia.

## Introduction

Sarcopenia is an age-related disease characterized by a loss of skeletal muscle mass and strength, as well as lower physical performance [1,2]. The prevalence of sarcopenia is 2.5% to 25.7% in Asian countries according to the Asian Working Group for Sarcopenia 2014 (AWGS2014) criteria [3]. Sarcopenia causes an increased risk of falls, fractures, morbidity, and even mortality [4]. However, it has not received sufficient attention and many have been misdiagnosed.

Sarcopenia has been reported to be associated with some cardiovascular diseases, especially heart failure (HF) [5]. The incidence of sarcopenia in elderly HF patients was higher than that in patients without HF [6]. The causal relationship between sarcopenia and HF is not yet clear, or they are mutually causal. Both skeletal muscle and myocardium are striated muscles; it is currently unclear whether the loss of skeletal muscle mass (SMM) is accompanied by a decrease in heart mass and function. Recent evidence suggests that pathological changes of SMM may reduce the effects of cardiac protective factors [7,8]. In experiments of animal models, it is observed that Akt protein kinase B is secreted by the skeletal muscle [9]. Akt protein kinase B is known as cardiac protective factors and may reduce cardiac injury [10]. Decreased Akt protein kinase B from muscle may reduce heart protective effects. Current evidence on the trend of changes in myocardial mass in patients with sarcopenia is insufficient. Only a few experiments have been conducted to observe the correlation between sarcopenia and myocardial mass. Heart atrophy occurs in people following bed rest or inactivity, which are known as risk factors for sarcopenia and physical injury [11,12,13]. In a study of community-dwelling older adults without cardiovascular disease, SMM was independently associated with left ventricular mass (LVM) [14]. HF may influence SMM through inflammation, oxidative stress, low ventricular ejection fraction, malnutrition, and lack of exercise [15,16]. These studies suggest that muscle mass and myocardium may be co-regulated during aging. In this study, the association of LVM with sarcopenia and its severity in hospitalized elderly were explored to validate this viewpoint.

## Methods

### Study design and subjects

One hundred seventy-four subjects aged ≥65 years and hospitalized in the Second Xiangya Hospital of Central South University were recruited in this study. According to the AWGS2019 criteria [3], low muscle mass is defined as appendicular skeletal muscle mass index (SMI) <7.0 kg/m^2^ in men and <5.4 kg/m^2^ in women when measured by dual-energy X-ray absorptiometry (DXA), or SMI <7.0 kg/m^2^ in men and <5.7 kg/m^2^ in women measured by bioelectrical impedance analysis (BIA); low muscle strength is handgrip strength <28 kg for men and <18 kg for women; low physical performance is 6-m walk <1.0 m/s or 5-time chair stand test ≥12 seconds. Sarcopenia is defined as low muscle mass, plus low muscle strength, and/or low physical performance. Coexistence of low muscle mass, low muscle strength, and low physical performance are diagnosed with severe sarcopenia. In this study, BIA was used to evaluate the SMM and 5-time chair stand test to assess physical performance. Based on AWGS2019 criteria, 71 patients were diagnosed with sarcopenia, 103 without sarcopenia in this study. Among the sarcopenic patients, 31 cases were non-severe and 40 were severe.

Clinical information, including age, gender, systolic blood pressure (SBP), diastolic blood pressure (DBP), pulse, and body mass index (BMI) were collected. Complete blood cell count (CBC), glucose, lipids, liver and kidney function, and C-reactive protein (CRP) were tested in the Department of the Clinic Laboratory.

Subjects with following diseases or conditions were excluded in this study: (1) unwilling to provide informed consent; (2) severe hepatic or renal dysfunction; (3) acute infection; (4) autoimmune diseases; (5) malignant tumor; (6) severe osteoarthritis or disability; (7)severe cardiovascular disease, including New York Heart Association class III or IV; (8) cognition impairment, psychiatric disorders, or poor cooperation. The study was approved by the institutional ethics committee of the Second Xiangya Hospital.

### Bioelectrical impedance analysis

BIA was performed with Inbody S10 by a trained nurse following the standardized procedure. Previous studies indicated that this eight-polar tetrapolar tactile system provides highly accurate results that strongly correlate with those obtained via DXA [17]. To obtain reliable measurements, alcohol was used to remove secretions and peeling skin cells before electrodes were placed. None of the subjects had fever, excessive fluid rehydration, or dehydration. When BIA was measures with Inbody S10, weight, fat mass, fat distribution, appendicular lean mass (ALM), SMM, abdominal circumference, and basal metabolic rate were calculated automatically.

### Handgrip strength

A hand-held dynamometer (CAMRY MODEL EH101, HaNDCReW, Guangdong, China) was used to evaluate the grip strength of the dominant hand. The subject stood, arms naturally drooping and gripping the dynamometer with full force. The test was repeated three times with a 30-second rest between each test, and the highest data was recorded.

### 5-time chair stand test

A chair about 48 cm away from the ground without armrests and a backrest was placed against the wall in a spacious room. The patients folded their arms around chest and performed a ‘stand up and sit down’ motion 5 consecutive times as fast as possible without the help of upper limb strength, recording the required time. The test was terminated if the patient could not complete this activity. The average time of three tests, with an interval of 10 minutes, was taken.

### Activities of daily living

The daily living ability of each subject was measured using activities of daily living (ADL) [18]. ADL include eating, dressing, personal hygiene, self-bathing, bowel control, bladder control, using the toilet, chair/bed transfer, mobility, and stair climbing. According to the degree to which participants could complete the 10 activities, investigators gave 0, 5, and 10 points. The total score ≤ 20, 25–70, and 75–100 points are classified as ‘totally dependent,’ ‘partially dependent,’ and ‘self-care,’ respectively.

### Echocardiography and left ventricular mass

Echocardiography was performed by an ultrasonography operator using a 2–4 MHz probe-equipped machine and reviewed by two ultrasonography experts. In each subject, standard echocardiography, which included two dimensional M-mode pulse Doppler and tissue Doppler imaging, was performed; the measurements were averaged over three cardiac cycles and adjusted for RR intervals. The left ventricular diameter (LVD), left atrial diameter (LAD), right ventricular diameter (RVD), right atrial diameter (RAD), aorta diameter (AO), pulmonary artery diameter (PA), interventricular septum diastolic thickness (IVSD), left ventricular posterior wall diastolic thickness (LVPWD), left ventricular ejection fraction (LVEF), and left ventricular fractional shortening(LVFS) were measured. LVM was calculated using the equation: LVM (g) = 0.8 × 1.04 × [(LVD + IVS + LVPW)^3^ – LVD^3^] + 0.6 [19].

### Mini-nutritional assessment-short form

The nutritional status of each subject was measured using mini-nutritional assessment-short form (MNA-SF) [20]. MNA-SF included six questions: (1) eating less food in the past 3 months due to issues with digestion, swallowing, chewing, or loss of appetite (0 = severe, 1 = mild to moderate, 2 = none); (2) weight loss during the past 3 months (0 = weight loss ≥3 kg, 1 = unconfirmed, 2 = weight loss of 1–3 kg, 3 score = none); (3) mobility (0 = bed or wheelchair, 1 = get up from bed or wheelchair, 2 = outdoor activities); (4) exposure to psychological stress or acute disease in the last 3 months (0 = yes, 1 = no); (5) neuropsychological problems (0 = severe senile dementia or depression, 1 = mild, 2 = none); (6) BMI (0 =< 19, 1 = 19–21, 2 = 21–23, 3 = ≥ 23). A total score of < 7, 8–11, or 12–14 points is classified as ‘malnourished,’ ‘at risk of malnutrition,’ and ‘normal nutritional status,’ respectively.

### Statistical analysis

The SPSS software program 26.0 was used for the statistical analysis. The traits of the subjects were expressed as mean ± standard deviation (SD), median (interquartile interval), or number (proportion). The Student’s t-test was employed to determine the distinction between two groups, and ANOVA was used to examine continuous variables among groups. The Wilcoxon rank sum test was utilized for non-parametric data. The Pearson correlation coefficient and multivariate linear regression model were used to examine relationships between continuous variables. A receiver-operating characteristic (ROC) curve was constructed, the area under the ROC curve was calculated, and threshold values were estimated to detect the sensitivity and specificity of LVM and BMI for the diagnosis of sarcopenia. *P* value <0.05 was statistically significant.

## Results

### Basic characteristics

A total of 174 subjects were enrolled in this study, including 71 sarcopenia (sarcopenia group) and 103 non-sarcopenia (non-sarcopenia group) patients. Among those with sarcopenia, 31 had non-severe sarcopenia and 40 had severe sarcopenia. The baseline characteristics of these two groups are shown in Table 1. There were no significant differences in age, sex, SBP, DBP, pulse, ADL, 5-time chair stand test, number of diabetes mellitus, ever smokers and drinkers, glucose, CRP, lipoprotein, or the indices of liver and renal functions between the two groups. Height, BMI, MNA-SF scores, instances of hypertension, and uric acid (UA) were lower in the sarcopenia group than in the non-sarcopenia group. In BIA measurements, the muscle mass of arms, legs, and trunk, total SMM, ALM, SMI, abdominal circumference, basal metabolic rate, and handgrip strength were lower in the sarcopenia group than in the non-sarcopenia group (Table 2). In echocardiography measurements, LVD, AO, PA, IVSD, LVPWD, and LVM were lower in the sarcopenia group than in the non-sarcopenia group. There were no significant differences in other BIA and echocardiography parameters between the two groups (Table 2).

**Table 1:** Clinic characteristics of the subjects.


VARIABLES	NON-SARCOPENIA (n = 103)	SARCOPENIA (n = 71)	*p* VALUE

Age (years)	80.01 ± 5.31	81.47 ± 6.54	0.108

Gender (male, %)	54 (52.43)	33 (46.48)	0.441

Height (cm)	160 (155, 166)	156 (150, 163)	0.002

BMI (kg/m^2^)	25.00 (23.20, 26.60)	21.90 (19.60, 23.60)	<0.001

SBP (mmHg)	139.74 ± 16.53	134.76 ± 17.59	0.059

DBP (mmHg)	74.09 ± 10.66	74.39 ± 10.28	0.851

Pulse (beats/min)	73.43 ± 12.15	74.45 ± 12.06	0.584

ADL	95 (85, 100)	95 (90, 100)	0.908

MNA-SF scores	13 (13, 14)	12 (11, 13)	<0.001

Smoker (%)	26 (25.24)	21 (29.58)	0.527

Drinker (%)	28 (27.18)	17 (23.94)	0.631

Hypertension (%)	87 (84.47)	48 (67.61)	0.009

Diabetes (%)	40 (38.83)	25 (35.21)	0.627

WBC (10^9^/L)	5.74 (4.79, 6.50)	5.49 (4.53, 6.81)	0.949

Hb (g/L)	124.55 ± 15.64	120.16 ± 15.69	0.072

PLT (10^9^/L)	177.00 (137.00, 217.00)	188.00 (151.00, 230.00)	0.186

ALT (μ/L)	14.50 (10.70, 17.50)	13.80 (9.70, 20.30)	0.818

AST (μ/L)	18.40 (15.90, 22.10)	20.80 (16.90, 25.50)	0.170

Cr (μmol/L)	73.10 (62.40, 92.80)	71.70 (60.00, 101.50)	0.560

UA (μmol/L)	341.67 ± 79.34	311.06 ± 91.66	0.021

FPG (mmol/L)	5.60 (5.03, 6.20)	5.70 (4.80, 6.40)	0.631

CRP (mmol/L)	8.32 (2.68, 8.32)	3.52 (2.13, 10.33)	0.865

TG (mmol/L)	1.38 (0.90, 1.81)	1.26 (0.84, 1.65)	0.237

LDL-C (mmol/L)	2.50 ± 0.79	2.44 ± 0.80	0.675


Data are expressed as the means ± standard deviation for normally distributed data and median (interquartile range) for nonnormally distributed data. Abbreviations: BMI, body mass index; SBP, systolic pressure; DBP, diastolic pressure; ADL, activities of daily living; WBC, white blood cell; Hb, hemoglobin; PLT, blood platelet count; ALT, alanine transaminase; AST, aspartate aminotransferase; Cr, creatinine; UA, uric acid; FPG, fasting plasma glucose; CRP, C-reactive protein; TG, triglyceride; LDL-C, low density lipoprotein cholesterol.

**Table 2:** BIA and echocardiography parameters in the subjects.


VARIABLES	NON-SARCOPENIA (n = 103)	SARCOPENIA (n = 71)	*p* VALUE

Total skeletal muscle mass (kg)	23.90 (20.50, 26.50)	18.40 (16.50, 22.40)	<0.001

Lean right arm (kg)	2.24 ± 0.49	1.76 ± 0.46	<0.001

Lean left arm (kg)	2.21 ± 0.47	1.74 ± 0.45	<0.001

Lean trunk (kg)	19.30 (16.80, 21.60)	15.60 (14.30, 18.80)	<0.001

Lean right leg (kg)	6.74 (5.71, 7.85)	4.94 (4.25, 6.57)	<0.001

Lean left leg (kg)	6.77 (5.79, 7.73)	5.04 (4.29, 6.57)	<0.001

ALM (kg/m^2^)	18.25 (15.28, 20.69)	13.18 (11.43, 17.66)	<0.001

SMI (kg/m^2^)	7.10 (6.20, 7.70)	5.60 (5.00, 6.60)	<0.001

Abdominal circumference (cm)	82.30 (75.50, 89.20)	76.80 (71.20, 83.80)	0.001

basal metabolic rate (KJ)	1327.13 ± 146.25	1160.09 ± 143.87	<0.001

Percent Body Fat (%)	30.80 (25.70, 36.80)	30.50 (25.20, 35.00)	0.545

Handgrip (kg)	24.04 ± 7.06	20.16 ± 6.64	<0.001

5-time chair stand test (s)	15.48 (11.77, 19.78)	14.81 (12.09, 20.07)	0.599

LVD (mm)	45.00 (43.00, 47.00)	42.00 (40.00, 46.00)	<0.001

LAD (mm)	34.00 (30.00, 37.00)	33.00 (29.00, 36.00)	0.079

RVD (mm)	28.35 ± 2.50	27.81 ± 3.00	0.206

RAD (mm)	28.00 (26.00, 30.00)	27.00 (26.00,30.00)	0.699

AO (mm)	32.00 (30.00, 35.00)	31.00 (28.00, 34.00)	0.037

PA (mm)	21.00 (20.00, 22.00)	20.00 (20.00, 22.00)	0.004

IVSD (mm)	10.00 (9.00,11.00)	9.5.00 (9.00, 10.00)	0.002

LVPWD (mm)	10.00 (9.00, 11.00)	9.00 (8.00, 10.00)	0.002

EF (%)	62.00 (60.00, 63.00)	62.00 (60.00, 63.00)	0.482

FS (%)	33.00 (31.00, 34.00)	32.00 (30.00, 34.00)	0.097

LVM (g)	157.06 (132.74, 176.04)	127.66 (109.69, 153.24)	<0.001


Data are expressed as the means ± standard deviation for normally distributed data and median (interquartile range) for nonnormally distributed data. Abbreviations: ALM, appendicular lean mass; SMI, skeletal muscle mass index; LVD, left ventricular diameter; LAD, left atrial diameter; RVD, right ventricular diameter; RAD, right atrial diameter; AO, aorta diameter; PA, pulmonary artery diameter; IVSD, interventricular septum diastolic thickness; LVPWD, left ventricular posterior wall diastolic thickness; EF, left ventricular ejection fraction; FS, left ventricular fractional; LVM, left ventricular mass.

### Comparison among non-sarcopenia and different extent of sarcopenia

Further analysis showed that there were no significant differences among the non-sarcopenia, non-severe sarcopenia and severe sarcopenia patients in sex, instances of hypertension, LAD, RVD, AO, RAD, EF, or FS. Compared with the non-sarcopenia group, higher age was observed in the severe sarcopenia group. Height, total SMM, muscle mass of arms, trunk, and legs, ALM, SMI, abdominal circumference, handgrip strength, PA, IVSD, and LVM were significantly lower in the severe sarcopenia group than in the other two groups (Table 3).

**Table 3:** Comparison among non-sarcopenia and different extent of sarcopenia.


VARIABLES	NON-SARCOPENIA (n = 103)	NON-SEVERE SARCOPENIA (n = 31)	SEVERE SARCOPENIA (n = 40)

Age (years)	80.01 ± 5.31	79.81 ± 5.10	82.75 ± 7.27*^#^

Gender (male, %)	54 (52.43)	17 (54.84)	16 (40.00)

Height (cm)	160 (155, 166)	158 (153, 165)	154 (147, 162)*

BMI (kg/m^2^)	25.00 (23.20, 26.60)	21.40 (20.10, 23.60)*	22.00 (18.63, 23.05)*

MNA-SF scores	13 (13, 14)	12 (11, 14)*	12 (10, 13)*^#^

Hypertension (%)	87 (84.47)	21 (67.74)	27 (67.50)*

Total skeletal muscle mass (kg)	23.75 ± 4.00	19.79 ± 3.76*	18.85 ± 3.88*

Lean right arm (kg)	2.24 ± 0.49	1.83 ± 0.43*	1.72 ± 0.48*

Lean left arm (kg)	2.21 ± 0.47	1.82 ± 0.43*	1.68 ± 0.47*

Lean trunk (kg)	19.23 ± 2.94	16.96 ± 2.74*	15.90 ± 3.09*

Lean right leg (kg)	6.74 (5.71, 7.85)	5.98 (4.75, 6.93)*	4.69 (4.06, 6.47)*

Lean left leg (kg)	6.77 (5.79, 7.73)	5.80 (4.66, 6.85)*	4.63 (4.10, 6.46)*

ALM (kg/m^2^)	18.25 (15.28, 20.69)	16.03 (12.00, 18.06)*	12.36 (11.08, 17.27)*

SMI (kg/m^2^)	7.10 (6.20, 7.70)	6.20 (5.10, 6.70)*	5.35 (4.93, 6.38)*

Abdominal circumference (cm)	82.30 (75.50, 89.20)	78.30 (73.00, 85.70)	75.90 (71.00, 81.63)*

Handgrip (kg)	24.00 (19.00, 28.00)	24.00 (19.00, 30.00)	16.00 (13.00, 22.00)*^#^

LVD (mm)	45.00 (43.00, 47.00)	42.00 (40.00, 47.00)	42.00 (39.25, 45.00)*^#^

LAD (mm)	34.00 (30.00, 37.00)	31.00 (30.00, 36.00)	33.00 (29.00, 37.00)

RVD (mm)	28.35 ± 2.50	27.87 ± 3.27	27.77 ± 2.85

RAD (mm)	28.00 (26.00, 30.00)	28.00 (26.00, 30.00)	27.00 (26.00, 29.75)

AO (mm)	32.00 (30.00, 35.00)	32.00 (29.00, 34.00)	30.00 (27.25, 34.75)

PA (mm)	21.00 (20.00, 22.00)	21.00 (20.00, 22.00)	20.00 (20.00, 21.75)*

IVSD (mm)	10.00 (9.00, 11.00)	10.00 (9.00, 10.00)*	9.50 (8.00, 10.75)*

LVPWD (mm)	10.00 (9.00, 11.00)	9.00 (9.00, 10.00)*	9.00 (8.00, 10.00)*

EF (%)	62.00 (60.00, 63.00)	61.00 (60.00, 63.00)	62.00 (60.00, 63.00)

FS (%)	33.00 (31.00, 34.00)	31.00 (30.00, 33.00)	32.00 (30.00, 34.00)

LVM (g)	157.06 (132.74, 176.04)	132.11 (114.13, 153.24)	123.02 (103.43, 153.50)*^#^


Data are expressed as the means ± standard deviation for normally distributed data and median (interquartile range) for nonnormally distributed data. *p < 0.05, compared with the non-sarcopenia group. # *p* < 0.05, compared with the non-severe sarcopenia group. Abbreviations: BMI, body mass index; ALM, appendicular lean mass; SMI, skeletal muscle mass index; LVD, left ventricular diameter; LAD, left atrial diameter; RVD, right ventricular diameter; RAD, right atrial diameter; AO, aorta diameter; PA, pulmonary artery diameter; IVSD, interventricular septum diastolic thickness; LVPWD, left ventricular posterior wall diastolic thickness; EF, left ventricular ejection fraction; FS, left ventricular fractional; LVM, left ventricular mass.

### Relationships between clinical variables, echocardiography parameters and SMM or SMI

To investigate whether age, height, BMI, MNA-SF scores, or echocardiography parameters were correlated with SMM or SMI, Spearman’s correlation was used to analyze the total study subjects (n = 174). The results revealed that height, BMI, MNA-SF scores, LVD, RVD, IVSD, LVPWD, and LVM were positively correlated with SMM; height, BMI, MNA-SF scores, LVD, LAD, RVD, IVSD, LVPWD, and LVM were positively correlated with SMI. No significant correlations were found between age, LAD, RAD, EF, FS, and SMM; SMI was not associated with age, RAD, EF, and FS (Table 4).

**Table 4:** Correlation between echocardiography parameters and SMM or SMI.


VARIABLES	SMM		SMI	
	
	r	*p* VALUE	r	*p* VALUE

Age	0.063	0.409	0.037	0.624

Height	0.501	<0.001	0.381	<0.001

BMI	0.452	<0.001	0.616	<0.001

MNA-SF scores	0.356	<0.001	0.460	<0.001

LVD	0.434	<0.001	0.459	<0.001

LAD	0.170	0.025	0.202	0.008

RVD	0.303	<0.001	0.276	<0.001

RAD	0.186	0.014	0.159	0.036

IVSD	0.232	0.002	0.244	0.001

LVPWD	0.243	0.001	0.257	0.001

EF	0.029	0.708	0.005	0.944

FS	0.009	0.903	0.050	0.515

LVM	0.416	<0.001	0.441	<0.001


Abbreviations: BMI, body mass index; ALM, appendicular lean mass; SMI, skeletal muscle mass index; LVD, left ventricular diameter; LAD, left atrial diameter; RVD, right ventricular diameter; RAD, right atrial diameter; IVSD, interventricular septum diastolic thickness; LVPWD, left ventricular posterior wall diastolic thickness; EF, left ventricular ejection fraction; FS, left ventricular fractional; LVM, left ventricular mass.

A multivariate regression analysis was further performed to evaluate the association between height, BMI, MNA-SF scores, LVM and SMM or SMI, parameters with no statistical difference and little correlation were excluded. Meanwhile, LVM was calculated from echocardiography parameters, so cardiac sizes were not included in the multivariate regression analysis. The results showed that height, BMI and LVM were independent factors that significantly influenced SMM and SMI (Table 5). Because there are multiple variables influencing LVM, a multivariate regression analysis was used to assess association between clinical variables and LVM. The results revealed that except BMI, age, gender, height, SBP, and Abdominal circumference found no significant correlations with LVM (Supplementary Table S1).

**Table 5:** Multivariate regression analysis of the association between BMI, MNA-SF scores, LVM and SMM or SMI.


VARIABLES	SMM (R^2^ = 0.475, *p* < 0.001)		SMI (R^2^ = 0.535, *p* < 0.001)	
	
	BETA	*p* VALUE	BETA	*p* VALUE

Constant	–19.059(–26.376, –11.742)	<0.001	–3.283(–5.000, –1.566)	<0.001

Height	0.162(0.123, 0.201)	<0.001	0.030(0.021, 0.039)	<0.001

BMI	0.378(0.190, 0.565)	<0.001	0.152(0.108, 0.196)	<0.001

MNA-SF scores	0.279(–0.124, 0.683)	0.174	0.070(–0.024, 0.165)	0.144

LVM	0.020(0.006, 0.034)	0.005	0.004(0.001, 0.007)	0.001


Abbreviations: BMI, body mass index; LVM, left ventricular mass.

### The ROC of LVM and BMI in predicting the presence of sarcopenia

**Figure 1:**
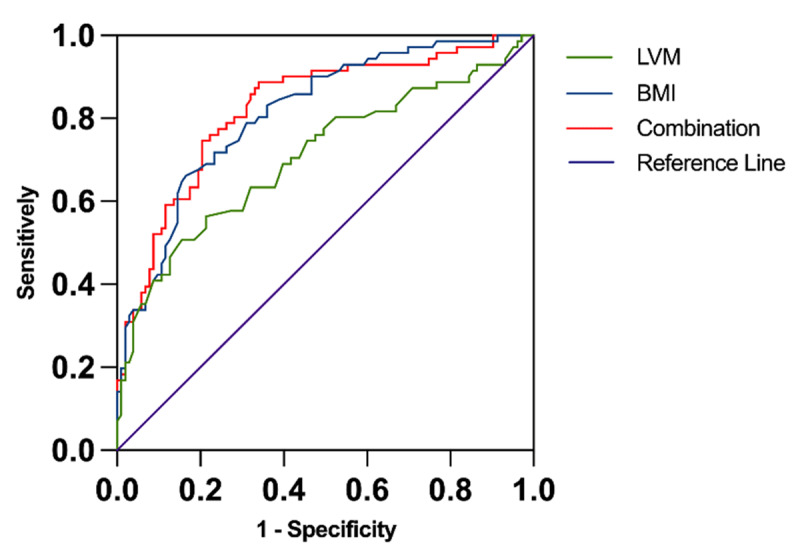
ROC analysis of LVM and BMI values in predicting the presence of sarcopenia.

ROC curve analysis was used to explore the predictive value of height, LVM, and BMI in the presence of sarcopenia. A cut-off height value of 158.75 cm was determined to predict the presence of sarcopenia with 63.1% sensitivity and 62% specificity (AUC: 0.635; 95% CI: (0.550, 0.720); *p* = 0.002). A cut-off LVM value of 127.73 g was determined to predict the presence of sarcopenia with 84.5% sensitivity and 50.7% specificity (AUC: 0.707; 95% CI: (0.626, 0.789); *p* < 0.001). A cut-off BMI value of 22.45 kg/m^2^ was found to predict sarcopenia with 83.5% sensitivity and 66.2% specificity (AUC: 0.814; 95% CI: (0.751, 0.878); *p* < 0.001). Because the area under curve of height was less than 0.7, LVM and BMI were used to predict sarcopenia. The sensitivity and specificity of the combined measurement of LVM and BMI in predicting sarcopenia were 66.0% and 88.7%, respectively (AUC: 0.825; 95% CI: (0.761, 0.889); *p* < 0.001) (Figure 1).

## Discussion

Among hospitalized elderly patients with normal left ventricular function, our main findings showed that sarcopenic patients had smaller left ventricular sizes and LVM. Body fat percentages were similar between both groups because non-sarcopenic groups had higher skeletal muscle mass, so the BMI will be higher in non-sarcopenic groups. It didn’t necessarily affect fat percentage, so there was no significant difference in body fat percentage between the two groups in the study.

Univariate regression analysis showed that BMI, MNA-SF scores, LVD, RVD, IVSD, LVPWD, and LVM were positively correlated with SMM; BMI, MNA-SF scores, LVD, LAD, RVD, IVSD, LVPWD, and LVM were positively correlated with SMI. In multivariate regression analysis, SMM and SMI were significantly correlated with LVM and BMI. The combination of LVM and BMI predicts sarcopenia with 66.0% sensitivity and 88.7% specificity (AUC: 0.825).

As aging diseases, sarcopenia and cardiovascular disease have common risk factors, such as age, obesity, lack of exercise, insulin resistance, and metabolic syndrome [15,21]. Heart disease may cause sarcopenia through common pathogenetic pathways, including inflammation, insulin-like growth factor-1, angiotensin, sex hormones, myostatin, physical inactivity, low EF, and malnutrition [15,16]. The Korea National Health and Nutrition Examination Survey reported that sarcopenia is associated with an increased risk of cardiovascular diseases [22]. It is well known that sarcopenia is closely related to cardiac disease in HF patients who develop secondary sarcopenia [23,24]. But, few studies have investigated the relationship between sarcopenia and cardiac structure and function without severe heart failure. We studied this relationship in hospitalized elderly patients without cardiac dysfunction and found SMM and SMI were strong and positively associated with LVD and LVM; and LVD and LVM were positively correlated with the severity of sarcopenia. In a study of a community population without cardiac disease, sarcopenic subjects had smaller left ventricular size and LVM than non-sarcopenic ones [14]. LVM was positively correlated with ALM in sarcopenic patients [25]. Our results are consistent with previous studies [14,25] and have confirmed this correlation in inpatients. However, some studies have obtained different results; for example, in a large cohort of Korean adults study, an increase in SMI was associated with a decrease in LVM index [26]. This discrepancy may attribute to the age of the population, ethnicity, and different research groups.

Changes of LVM have traditionally been considered a clinically adverse phenomenon of cardiac function, such as left ventricular hypertrophy (LVH). LVH is associated with higher cardiovascular risk and poorer prognosis [27]. But, LVH as a secondary compensatory mechanism can increase heart work, such as the athlete’s heart [28]. In our study, LVM was positively correlated with SMM, and SMM was not associated with EF and FS (left ventricular function). Therefore, we hypothesized that the sarcopenia associated with cardiac atrophy may be an aging change and also an impact on myocardium, resulting in a similar sarcopenia process within the myocardium. The observed reduction in LVM may represent myocyte loss that occurs in conjunction with skeletal muscle loss as a systemic manifestation that extends to key organs, such as the heart. Wasting changes in skeletal muscle were described among patients with advanced heart failure [29]. A variety of systemic and humoral mechanisms have been suggested for possible interactions between skeletal muscle and the diseased heart [30,31,32]. In our study, although some patients had heart and chronic diseases, such as coronary artery disease, hypertension, and diabetes mellitus that can lead to myocardial hypertrophy and heart enlargement, a positive correlation between LVM and SMM was still found.

In multivariate regression analysis, LVM and BMI were independent factors that significantly influenced the SMM in this study. It provides a clue that LVM and BMI may be associated with skeletal muscle wasting. Further analysis found single LVM and BMI measurements had relatively high sensitivity but low specificity, while combination measurement had a higher specificity, for predicting sarcopenia.

There are two main limitations in this study. Firstly, the influence of multimorbidity on LVM and cardiac structure cannot be excluded completely. There is no difference in baseline characteristics in sarcopenia and non-sarcopenia patients, so confounding influencing factors are eliminated and the results are representative to some extent. Secondly, the limited number of subjects and the cross-sectional nature of this study do not allow drawing conclusions about the nature of the relationship. Larger cohort studies are needed to further clarify the impact of sarcopenia on heart structure and function, and vice versa.

## Conclusion

In hospitalized elderly patients, LVM was positively correlated with SMM and the severity of sarcopenia; LVM combined with BMI may be helpful in diagnosing sarcopenia.

## Appendix

**Supplementary Table S1:** Multivariate regression analysis of the association between clinical variables and LVM.

LVM (R^2^ = 0.229, *p* < 0.001)		

VARIABLES	BETA	*p* VALUE

Constant	–35.829 (–166.381, 94.723)	0.589

Age	0.499 (–0.438, 1.435)	0.295

Gender	–10.926 (–23.297, 1.445)	0.083

Height	0.302 (–0.173, 0.777)	0.212

SBP	0.038 (–0.404, 0.327)	0.836

DBP	0.197 (–0.395, 0.788)	0.512

BMI	5.116 (2.547, 7.684)	<0.001

Abdominal circumference	–0.344 (–1.267, 0.579)	0.463

Abbreviations: LVM, left ventricular mass; SBP, systolic pressure; DBP, diastolic pressure; BMI, body mass index.
